# Dose–volume parameters of MRI-based active bone marrow predict hematologic toxicity of chemoradiotherapy for rectal cancer

**DOI:** 10.1007/s00066-020-01659-z

**Published:** 2020-07-03

**Authors:** Łukasz Kuncman, Konrad Stawiski, Michał Masłowski, Jakub Kucharz, Jacek Fijuth

**Affiliations:** 1 Department of Radiotherapy, Medical University of Lodz, Zakład Radioterapii, W.W.C.O.iT. im. M. Kopernika w Łodzi, Pabianicka 62 street, 93-513 Łódź, Poland; 2 Department of External Beam Radiotherapy, Regional Cancer Center, Copernicus Memorial Hospital of Lodz, Zakład Teleradioterapii, Wojewódzkie W.W.C.O.iT. im. M. Kopernika w Łodzi, Pabianicka 62 street, 93-513 Łódź, Poland; 3 Department of Biostatistics and Translational Medicine, Medical University of Lodz, Zakład Biostatystyki i Medycyny Translacyjnej, Cenrum Mazowiecka 15 street, 92-215 Łódź, Poland; 4 Department of Uro-Oncology, Maria Sklodowska-Curie Memorial Cancer Centre and Institute of Oncology Warsaw, Klinika Nowotworów Układu Moczowego, Centrum Onkologii-Instytut im. Marii Skłodowskiej—Curie ul. Roentgena 5, 02-781 Warszawa, Poland

**Keywords:** Bone marrow sparing radiotherapy, Lymphopenia, Magnetic resonance imaging, Immune system, Immunosuppression

## Abstract

**Purpose:**

Magnetic resonance imaging (MRI) is routinely used for locoregional staging of rectal cancer and offers promise for the prediction of hematologic toxicity. The present study compares the clinical utility of MRI-based active bone marrow (BMact) delineation with that of CT-based bone marrow total (BMtot) delineation for predicting hematologic toxicity.

**Methods:**

A prospective cohort study was performed. Eligible patients had stage II/III rectal cancer and qualified for preoperative chemoradiotherapy. The BMact areas on T1-weighted MRI were contoured. The impact of the dose–volume parameters of BMact/BMtot and clinical data on hematologic toxicity were assessed. Basic endpoints were the occurrence of grade 3/4 hematologic toxicity and peripheral blood parameters reaching a nadir. Linear regression models were generated for the nadirs and receiver operating characteristic (ROC) curves for the occurrence of grade 3/4 hematologic toxicity.

**Results:**

Thirty-five patients were enrolled. Women presented higher dose–volume parameters of BMact, BMtot, and lymphocyte nadir (ALCnadir%) than men. Models for the prediction of ALCnadir% (V5-V20BMtot, V5-V30BMact) and platelet nadir (PLTnadir%; V5-V10BMtot, V5-V20BMact) were statistically significant. In the ROC curves, a baseline lymphocyte level of 1.81 × 10^3^/ml was adopted as the cutoff for predicting grade 3/4 lymphopenia, with specificity of 77.8% and sensitivity of 73.1%. The multivariate linear regression model for ALCnadir% had R^2^ = 0.53, *p* = 0.038. In the tenth step of selection, V5BMact (*p* = 0.002) and gender (*p* = 0.019) remained. The multivariate linear regression model for PLTnadir% had R^2^ = 0.20, *p* = 0.34. In the sixth step of selection, V15BMact remained (*p* = 0.026).

**Conclusion:**

The dose–volume parameters of BMact serve as better predictors of ALCnadir% and PLTnadir% than BMtot.

## Introduction

Rectal cancer is the seventh most common cancer among men and the tenth most common among women, with approximately 704,000 new cases and 301,000 deaths estimated in 2018 worldwide [1]. Preoperative chemoradiotherapy forms part of the multidisciplinary treatment protocol for stage II and III of the Union for International Cancer Control’s (UICC) classification rectal cancer [2].

The immune status of patients with cancer has gained prominence over recent years. Dividing cells, such as the immune system progenitors in active bone marrow, are prone to damage caused by ionizing radiation. As approximately half of the active bone marrow in adults is located in the pelvis, radiotherapy (chemoradiotherapy) of this region may result in hematologic toxicity [3]. On the other hand, radiotherapy may stimulate the immune system and augment the potential of immunotherapy [4]. As with other cancers, absolute lymphocyte count (ALC) before, during, and after radiotherapy (chemoradiotherapy) is known to serve as a predictive and prognostic marker in rectal cancer [5–7]. In addition, ALC can affect the incidence of complete clinical response, which allows implementation of a promising, cost-effective watch-and-wait strategy [6, 8].

Previous studies have found the dose–volume parameters of total bone marrow (BMtot) of the pelvis to be associated with hematologic toxicity arising as a result of radiotherapy or chemoradiotherapy of rectal cancer [3, 9]. In addition, studies based on positron-emission tomography (PET) have examined possible correlations between the dose–volume parameters of active bone marrow (BMact) in gynecological malignancies and anal canal cancer [10–15]. However, PET has limited value in the staging of rectal cancer [2].

Magnetic resonance imaging (MRI) is routinely used for locoregional staging of rectal cancer [2]. Additionally, MRI is arguably the most sensitive imaging modality for evaluating bone marrow [16]. Active and inactive bone marrow differ with regard to their chemical and cellular content: BMact (40% fat, 40% water, and 20% protein) has a slightly higher or similar signal to skeletal muscle in T1-dependent MRI images, while inactive marrow (80% fat, 15% water, and 5% protein) is hyperintensive [16–18]. MRI allows clear differentiation between hematologically active and inactive bone marrow [16]; it may therefore be useful in planning radiation therapy, as the obtained dose–volume constraints of BMact can be used to optimize treatment planning and reduce hematologic toxicity.

Its use in the pelvic region has previously been evaluated, but the only study did not analyze the findings with regard to BMtot [19]. Therefore, the aim of the present study was to assess the clinical utility of a T1-weighted MRI sequence as a tool for delineating active bone marrow in the pelvic region and to compare this method with objective CT-based delineation of total bone marrow (BMtot) by analyzing the relationship between the dose–volume parameters of BMact and BMtot and hematologic toxicity.

## Materials and methods

### Study design and participants

A prospective single-arm phase II cohort study was performed. The inclusion criteria for patients comprised histologically confirmed stage II and III TNM UICC rectal cancer, qualification to preoperative chemoradiotherapy, WHO/ECOG performance status 0–2, and age ≥18 years. Exclusion criteria included any contraindication to chemoradiotherapy, the presence of clinically significant cardiovascular history, renal or liver dysfunction, pregnancy, previous radiotherapy to the pelvic area, systemic cancer therapy (including neoadjuvant chemotherapy), or hematologic disorders. All patients provided written informed consent to take part. The study was approved by the national bioethical commission.

### Procedures

Staging and qualification to treatment were performed according to present NCCN guidelines and approved by a multidisciplinary cancer center board. All patients underwent radiotherapy planning CT in the prone position with 5 mm slice thickness.

Total bone marrow (BMtot) was defined as the volume limited by external contour of all bones in the pelvic region, visualized on CT, as proposed by Mell et al. [20]. This method restricts the influence of the width and level of the CT window on contouring [20]. The bones in the pelvic region were defined as the volume containing the hip bones, ischium, pubic bones, acetabulum, and proximal femur, from the upper border of the femoral heads to the lower border of the ischial tuberosity, the lumbosacral spine up to the height of the upper border of iliac crest.

**Fig. 1:**
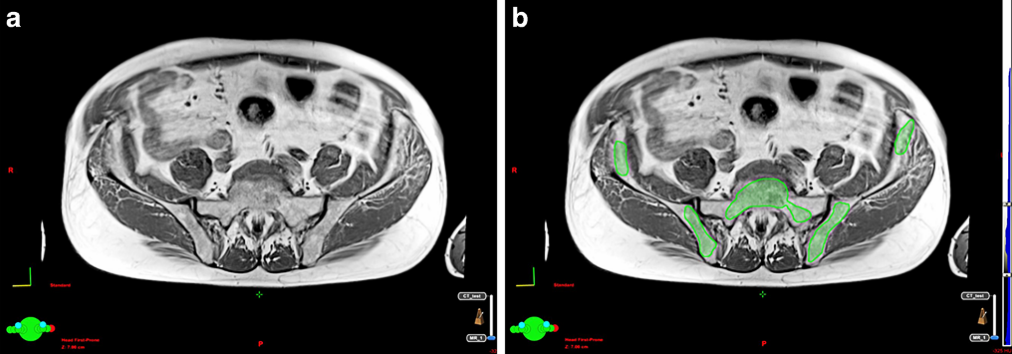
Contouring method of active bone marrow based on magnetic resonance imaging. **a** T1-weighted transverse MRI image, **b** T1-weighted transverse image with active bone marrow contour

In addition, all patients underwent a 1.5T MRI transverse T1-weighted sequence encompassing all pelvic bones, as described above (field of view [FOV] = 400 mm, matrix size 384 × 384, repetition time [TR] = 691 ms, echo time [TE] = 9 ms, slice thickness 5 mm, phase oversampling 50%; parameters were modified as required by MRI software in response to anatomy of the patient). This sequence was fused with radiotherapy planning CT scans. Fusion alignment was performed based on bone anatomy for active bone marrow (BMact) delineation. The T1-weighted sequence allows accurate determination of the cellular content of bone marrow to be performed [16, 21]. The fat tissue can be identified based on its short T1 relaxation time and high signal in T1-weighted sequence, and yellow (inactive) bone marrow can be contrasted with active bone marrow based on its high fat content [16, 21]. BMact demonstrates decreased signal intensity, i.e., lower than subcutaneous fat but higher than disk or muscle tissue. The areas in bones in the pelvic region with a signal intensity equal to or slightly higher than that of muscles on T1-weighted sequence were contoured as BMact, without using semi-automatic methods ([19]; Fig. 1). To minimize the subjectivity of the assessment, BMact contouring was performed by one radiation oncologist and revised by another. BMtot and BMact were not regarded as the organ at risk and the dose was not intentionally reduced.

All patients underwent 5‑fluorouracil-based chemoradiotherapy to 50.4–54 Gy in two stages. The elective target volume was delineated according to RTOG guidelines and irradiated to 45 Gy/1.8 Gy [22]. The mesorectum with the tumor, with a 2–5 cm margin, was irradiated to 50.4–54 Gy/1.8 Gy. Three cycles of two-day 5‑fluorouracil (400 mg/m^2^/d) with leucovorin (20 mg/m^2^/d) were administrated concurrently with radiotherapy every 14 days. Complete blood count samples were collected every week, and a renal and liver function test was performed every 2 weeks.

According to NCCN guidelines, the main irradiation technique was 3D conformal radiotherapy; dynamic irradiation techniques (intensity-modulated/arc radiotherapy) were reserved for unique clinical situations such as those associated with uncommon anatomy [2].

### Endpoints and statistical analysis

The size of the sample was estimated prospectively: 34 patients were required. The following assumptions were made for linear regression: statistical power of 80%; nine predictors (V5–V45); level of significance: alpha 0.05; effect size (defined as partial R2) = 0.4 (f2 = 0.65). Occurrence of grade 3/4 CTCAE (version 4) hematologic toxicity and peripheral blood parameter nadirs were adopted as basic endpoints. Nadirs were specified as percentage of baseline value (lymphocytes: ALCnadir%; neutrocytes: ANCnadir%; platelets: PLTnadir%; red blood cells: RBCnadir%). The following clinical data items, indicated to be potentially significant by previous studies, were included in the analysis: age, gender, body mass index (BMI), WHO/ECOG performance status, duration of chemoradiotherapy, use of arc irradiation techniques. Spearman’s rank correlation test or the Mann–Whitney U test was used to determine the relationship between clinical data and dose–volume parameters and hematologic toxicity, depending on the type of clinical data. Dose–volume parameters were V5-V45BMact and V5-V45BMtot, specified as volume of BMact and BMtot receiving 5 to 45 Gy, respectively (in percentages of whole volume of BMact or BMtot). Linear regression models were generated for each dose–volume parameter and the nadirs of blood count parameters (ALCnadir%, ANCnadir%, PLTnadir%, RBCnadir%). Additionally, ROC curves were generated for the occurrence of grade 3/4 hematologic toxicity. Cut-off was determined based on the Youden index.

Statistically significant results obtained in univariate analysis were included in multivariate linear regression models with stepwise selection using backward elimination. A stepwise selection model was used to minimize the predicted effect of collinearity of predictors. Probability was determined using the F test. The introductory value was 0.05, removal value was 0.1; *p*-values <0.05 were regarded as statistically significant. IBM SPSS version 25 (IBM Corp., Armonk, NY, USA) was used for calculations.

## Results

Thirty-five patients meeting the inclusion criteria, 18 men (M) and 17 women (W), were enrolled in the study. Of these, 33 had stage III rectal cancer and 2 patients had stage II rectal cancer according to TNM UICC 7th edition. All patients were in good performance status (28 ECOG‑0, 7 ECOG-1), aged 44–76 years old (median [Me] age 65 years), and without comorbidities preventing chemoradiotherapy. All patients completed the planned treatment; one patient did not reach half the middle cycle of concurrent chemotherapy due to infection not related to treatment. Time of treatment was 37 to 44 days (Me: 38 days) and was not associated with any blood parameter nadirs. None of the patients had used steroids during the study period.

### Clinical data

Median BMtot volume was 1632 ml, median BMact volume was 178 ml. The mean proportion of BMact/BMtot for the whole cohort was 10.9%. Older age was associated with a lower proportion of BMact/BMtot (R = 0.34, *p* < 0.05), but not with baseline or nadir levels of ALC, ANC, PLT, or RBC. No significant differences were found between genders regarding BMact/BMtot.

The female participants demonstrated lower BMtot (Me: 1340 ml vs. 1797 ml *p* < 0.001) and BMact volume (Me: 135 ml vs. 207 ml *p* < 0.001). Both weight and height were associated with BMtot (R = 0.55, *p* < 0.001) and BMact volume (R = 0.57, *p* < 0.001). No differences in PTV volume were found between genders. The women had poorer (higher) dose–volume parameters of BMtot than men (Table 1) and lower baseline RBC (RBC0; *p* < 0.05; Me 4.82 × 10^6^/ml vs. 4.58 × 10^6^/ml), baseline ALC (ALC0; *p* < 0.05; Me 1.64 × 10^3^/ml vs. 1.77 × 10^3^/ml), and baseline PLT (PLT0) scores (*p* < 0.01; Me 215 × 10^5^/ml vs. 270 × 10^5^/ml); however, no difference in baseline ANC (ANC0) was observed (*p* = 0.21). The women also had lower ALCnadir% (*p* < 0.01) than men.

**Table 1:** Dose–volume parameters of BMact and BMtot in men and women

	Women (*n* = 17)	Men (*n* = 18)	*p-*value
	Median (%)	Median (%)	
V5BMtot	92.5	88.2	0.035*
V10BMtot	84.6	82.7	0.062
V30BMtot	67.7	61.9	0.032*
V35BMtot	48.6	41.2	0.032*
V40BMtot	35.6	31.9	0.057

*Statistically significant *p*-value, *BMact* bone marrow active, *BMtot* bone marrow total, *V5-V40* volume (in percentages) receiving radiation dose of 5–40 Grey, respectively

Performance status was associated with RBC0 (*p* < 0.05; Me ECOG 0: 4.65 × 10^6^/ml, ECOG 1:4.36 × 10^6^/ml) and PLT0 (*p* < 0.05; Me ECOG 0: 253 × 10^5^/ml, ECOG 1:321 × 10^5^/ml). BMI was correlated with RBC0 (R = 0.41; *p* < 0.05) and RBCnadir% (R = −0.39; *p* < 0.05). No other associations were observed between performance status/BMI and blood count nadirs. Due to the low number of patients with stage II disease, no statistical analysis was performed regarding clinical cancer stage.

Twenty-six patients were irradiated using the static 3D conformal technique and nine using the arc technique (either in one or both stages). Lower dose–volume parameters were observed for both BMact and BMtot when using the arc technique, at medium and high doses (V20–V45).

All baseline blood parameters, including ANC0 (R = 0.59), ALC0 (R = 0.54), RBC0 (R = 0.67), and PLT0 (R = 0.83), were strongly associated with the respective nadirs expressed as absolute values: ANCnadir, ALCnadir, RBCnadir, PLTnadir (*p* < 0.001).

CTCAE grade 3 lymphopenia was observed in 26 patients (74.3%), while 9 patients (25.7%) had CTCAE grade 2 lymphopenia. No grade 2/3 thrombocytopenia was observed. ROC curves for the dose–volume parameters and significant clinical data were generated for the prediction of CTCAE grade 3 lymphopenia. ALC0 was predictive for the occurrence of CTCAE grade 3 lymphopenia and demonstrated a large AUC (AUC = 0.81; *p* = 0.007). A baseline lymphocyte value of 1.81 × 10^3^/ml was set as the cutoff for predicting grade 3 complications, with a specificity of 77.8% and sensitivity of 73.1%.

### Dose–volume parameters

Linear regression models were generated for each dose–volume parameter (V5-V45BMtot and V5-V45BMtot as predictive factors) for each nadir of blood parameters, ANCnadir%, ALCnadir%, RBCnadir%, and PLTnadir%, as dependent variables. Only models for the prediction of ALCnadir% (V5-V20BMtot, V5-V30BMact) and PLTnadir% (V5-V10BMtot, V5-V20BMact) were statistically significant (Table 2).

**Table 2:** Linear regression model parameters

	ALCnadir%		PLTnadir%	
	BMact	BMtot	BMact	BMtot
	*p-*value	*p-*value	*p-*value	*p-*value
V5	0.001*	0.001*	0.028*	0.049*
V10	0.003*	0.002*	0.022*	0.044*
V15	0.005*	0.003*	0.026*	0.06
V20	0.008*	0.023*	0.044*	0.121
V25	0.012*	0.071	0.1	0.226
V30	0.022*	0.121	0.161	0.265
V35	0.097	0.325	0.339	0.401
V40	0.216	0.333	0.289	0.306
V45	0.685	0.731	0.427	0.494

*Statistically significant *p*-value, *ALCnadir%* lymphocyte nadir (in percentages), *PLTnadir%* platelet nadir (in percentages), *BMact* bone marrow active, *BMtot* bone marrow total, *V5-V45* volume (in percentages) receiving radiation dose of 5–45 Grey, respectively

A strong correlation was observed between all respective dose–volume parameters of BMtot and BMact (V5BMact-V5BMtot … V45BMact-V45BMtot; *p* < 0.0001).

### Multivariate linear regression models

The statistically significant clinical data and dose–volume parameters identified in univariate analysis were included in multivariate linear regression models. Two separate models for predicting ALCnadir% and PLTnadir% were generated.

All statistically significant factors for ALCnadir% from univariate models (V5BMtot, V10BMtot, V15BMtot, V20BMtot, V5BMact, V10BMact, V15BMact, V20BMact, V25BMact, V30BMact, gender) were included in the multivariate regression model for ALCnadir%. The initial model had R^2^ = 0.53, F = 2.38, *p* = 0.038. In the tenth step of selection (R^2^ = 0.40) V5BMact and gender remained. Both variables were significant for the model: gender (*p* = 0.019, Beta(std) = 0.34 95%CI [0.92; 9.44]), V5BMact (*p* = 0.002, beta (std) = −0.48, 95%CI [−0.53; −0.14]). The findings are shown in greater detail in Table 3. All statistically significant factors for PLTnadir% from the univariate model (V5BMtot, V10BMtot, V5BMact, V10BMact, V15BMact, V20BMact) were included in the multivariate regression model for PLTnadir%. The initial model had R^2^ = 0.20, F = 1.19, *p* = 0.34. In the sixth step of selection (R^2^ = 0.14), V15BMact remained (*p* = 0.026), beta (std) = −0.38, 95%CI [−0.68; −0.046].

**Table 3:** Multivariate linear regression model for ALCnadir% (backward elimination method)

Model	R	R‑square	Adjusted R‑square	Change statistics	
				F change	Sig. F change
1	0.730^a^	0.533	0.309	2.38	0.038
2	0.729^b^	0.532	0.337	0.36	0.851
3	0.727^c^	0.529	0.360	0.146	0.706
4	0.724^d^	0.524	0.378	0.260	0.615
5	0.712^e^	0.507	0.379	0.931	0.343
6	0.687^f^	0.472	0.359	1.90	0.179
7	0.679^g^	0.461	0.368	0.623	0.436
8	0.653^h^	0.426	0.349	1.865	0.183
9	0.635^i^	0.404	0.346	1.17	0.288
10	0.633^j^	0.401	0.363	0.144	0.707

^a^Predictors (constant): gender, V5BMact, V10BMact, V15BMact, V20BMact, V25BMact, V30BMact, V5BMtot, V10BMtot, V15BMtot, V20BMtot

^b^Predictors (constant): gender, V5BMact, V10BMact, V20BMact, V25BMact, V30BMact, V5BMtot, V10BMtot, V15BMtot, V20BMtot

^c^Predictors (constant): gender, V5BMact, V10BMact, V20BMact, V25BMact, V5BMtot, V10BMtot, V15BMtot, V20BMtot

^d^Predictors (constant): gender, V5BMact, V10BMact, V25BMact, V5BMtot, V10BMtot, V15BMtot, V20BMtot

^e^Predictors (constant): gender, V5BMact, V10BMact, V25BMact, V10BMtot, V15BMtot, V20BMtot

^f^Predictors (constant): gender, V5BMact, V10BMact, V10BMtot, V15BMtot, V20BMtot

^g^Predictors (constant): gender, V5BMact, V10BMtot, V15BMtot, V20BMtot

^h^Predictors (constant): gender, V5BMact, V15BMtot, V20BMtot

^i^Predictors (constant): gender, V5BMact, V15BMtot

^i^Predictors (constant): gender, V5BMact

*ALCnadir%* lymphocyte nadir (in percentages), *BMact* bone marrow active, *BMtot* bone marrow total, *V5-V45* volume (in percentages) receiving radiation dose of 5–45 Grey, respectively

## Discussion

The present study set out to confirm whether MRI-based BMact contouring as an organ at risk has greater clinical utility than the CT-based BMtot contouring method in the prediction of hematologic toxicity. It was found that only lymphopenia reached CTCAE grade 2 or 3 and is therefore of greater clinical importance. The main advantages of contouring the entire volume of bone as a BMtot surrogate are that additional images are not needed, and that implementation is simpler. BMtot volume was found to be almost 10 times that of BMact in the present study. As the MRI-based BMact volume is smaller than BMtot obtained by CT, it could offer greater potential as an organ at risk for plan optimization during 3D/IMRT radiotherapy planning. In view of the above, recent studies have been able to delineate BMact using the following imaging modalities: 18F-fluorothymidine positron-emission tomography (FLT-PET) [10, 13], 18F-fluorodeoxyglucose positron-emission tomography (FDG-PET) [10–12, 14, 15, 23–25], and MRI [19].

Our results confirm that an association exists between the dose–volume parameters of pelvic BMtot and hematologic toxicity, as noted previously [26]. Our BMact results are partly consistent with the only study assessing MRI-based BMact contouring [19]. In our study, V5BMact was found to be predictive for lymphocyte nadirs (ALCnadir%) and V15BMact for platelet nadirs (PLTnadir%) in multivariate linear regression models. Wang et al. found dose–volume parameters from low dose ranges (V5BMact) to be associated with WBCnadirs and PLTnadirs in a multivariate linear regression model; however, the study used a different chemotherapy regimen based on oxaliplatin, which is currently not recommended in neoadjuvant chemoradiotherapy of rectal cancer [2], and delineated BMact in a different range than in other studies (PTV +2.5 cm) [19]. In the present study, both the BMact and BMtot dose–volume parameters were found to be associated with platelet and lymphocyte nadirs, which is similar to data obtained by PET-based BMact contouring. FDG-PET-based BMact and BMtot dose–volume parameters were found to be associated with hematologic toxicity in cervical and anal canal cancers, suggesting that BMact contouring could be a valuable method [11, 23]. However, similar studies regarding the dose–volume parameters of both BMtot and BMact and hematologic toxicity in anal canal cancer indicated that BMact parameters, defined by FDG-PET, failed to improve models [15].

As reports on the clinical value of BMact and BMtot contouring are conflicting, the present study identifies the most valuable predictors of hematologic toxicity, in order to avoid the effect of collinearity of variables (dose–volume parameters). MRI-based V5BMact remained in the final step of selection for the ALCnadir% multivariate regression model, and V15BMact in PLTnadir%. Noteworthily, the ALCnadir% model was significant, and V5BMact and V15BMact were significant for the ALCnadir% and PLTnadir% models. No such significance was reported in the previous studies.

Our results suggest that the dose–volume parameters of MRI-based BMact parameters may have greater clinical utility in predicting hematologic toxicity. The next stage of the research should be to determine the dose–volume constraints for active bone marrow to optimize treatment planning. The INTERTECC‑2 trial of a cohort of cervical cancer patients found bone marrow-sparing radiotherapy based on PET-BMact to demonstrate lower toxicity than CT-BMtot delineation; however, the authors note that reduction of toxicity could not be fully explained, because the PET-BMact sparing group had unintentionally also demonstrated better dose–volume BMtot parameters [12].

An additional part of the study was the analysis of clinical data affecting hematologic toxicity. Female gender is often reported as a risk factor for hematologic toxicity in rectal [3, 9, 19] and anal canal cancer [27]. However, it is unknown whether female gender is an independent risk factor for hematologic toxicity or if women demonstrate worse dose–volume parameters due to the different shape of the pelvis, thus being more prone to hematologic toxicity. Our data suggest that although there were no differences in PTV volume between genders, women demonstrated smaller BMact and BMtot volumes and, due to the spatial location, poorer dose–volume parameters. In addition, the multifactorial analysis performed in the present study also indicates that female sex is an independent risk factor.

Baseline blood parameters (ALC0, ANC0, PLT0, RBC0) are a strong factor associated with reduced nadirs in the present paper and several previous studies [28, 29]. An ALC0 level of 1.81 × 10^3^/ml allowed the occurrence of grade 3 CTCAE lymphopenia to be predicted with high sensitivity (73.1%) and specificity (77.8%). Similarly, in the largest prospective study accessing hematologic toxicity in prostate cancer patients undergoing radiotherapy, ALC0 ≤ 1.83 × 10^3^/ml allowed prediction of grade 3 CTCAE lymphopenia [28]. Many studies do not include any evaluation of hematologic toxicity baseline blood parameters in their analysis, and this could potentially influence the results. In view of our present findings and those of Sini et al., it seems justified to report nadirs as a proportion of initial value, as in the current study [28].

Finally, lymphocyte toxicity is gaining importance in the era of understanding the immune mechanisms responsible for cancer development and treatment. The relationship between radiotherapy and immunotherapy is under investigation, and the results of clinical trials assessing the combination of radiotherapy and immunotherapy have recently been published [30]. Lymphocytes play a key role in these mechanisms. In colorectal cancer, baseline ALC and the ratio of lymphocytes to neutrocytes is are prognostic factors affecting overall and progression-free survival [5, 31, 32]. It is important to note that almost three quarters of the patients included in the current study had grade 3 lymphopenia. In most randomized trials, lymphocyte count is not reported as a separate parameter and is included in whole white blood cell count [33]. Lymphocytes are extremely sensitive to ionizing radiation. Of all hematologic toxicities, lymphopenia occurs most frequently [3]. Grade 2 and 3 lymphopenia were observed in 97.5% of patients undergoing chemoradiotherapy for rectal cancer (grade 3 in 56.7%), compared to only 11.7% grade 2 leukopenia (no grade 3) [3].

The main limitation of our study is its small sample size; however, the number of enrolled patients is comparable to that of other studies examining BMact delineation (26 in Rose et al. [11], 45 in Rose et al. [15], 44 in Franco et al. [23], 17 in Elicin et al. [14], 35 in Mell et al. [12]) [11, 12, 14, 15, 23].

Although MRI is an accurate method for imaging bone marrow, it does not provide the numerical data useful to assessing it. Contouring of BMact is partly subjective. In addition, contouring of active bone marrow is a time-consuming procedure and requires some expertise. The validity of the method may be indirectly confirmed by the appearance of changes observed in the bone marrow under the influence of radiation [16]. Changes in the proportion of active and inactive bone marrow can be observed after even 8 days and can last for up to 2 years [16]. The next step in development may be to move towards semi-automated contouring of BMact [34]. Hence, the present study assesses whether contouring of active bone marrow based on MRI, which, in principle, is partly subjective, may be clinically useful.

Patients with rectal cancer were included in the prospective selection of the study cohort for three reasons: lymph node irradiation and MRI are routinely used in this indication, and 5‑fluorouracil-based chemotherapy has low myelotoxic potential. The chemotherapy regimen with low myelotoxic potential minimizes the effect of this treatment on hematologic toxicity in combination chemoradiotherapy. This enables more objective assessment of the effect of radiation therapy on myeloid toxicity. Furthermore, supportive therapies (transfusions, granulocyte growth factors) and treatment interruptions are more frequently observed during myelotoxic chemotherapy regimens, which can affect study endpoints. However, there remain some doubts as to what extent the results of this study can be translated to other than 5‑fluorouracil chemoradiotherapy regimens. Patients undergoing radiotherapy/chemoradiotherapy after surgery procedures (endometrial, rectal, prostate cancer) were excluded due to the possible impact of surgery on peripheral blood parameters associated with perioperative and postoperative complications. In addition, MRI is rarely performed in these patients. Prostate cancer patients were not included due to conflicting data on pelvic lymph node irradiation.

Nevertheless, this is the first study to compare the objective method of BMtot contouring with the promising but partly subjective MRI-BMact contouring method. Our study cohort was homogenous and was not affected by surgery. In addition, the employed chemotherapy regimen has less myelotoxic potential than those including cisplatin or mitomycin in cervical and anal cancer patients. Another strength of our study is its use of MRI, which, unlike PET-CT, is routinely used in the diagnosis of pelvic tumors.

## Conclusion

The dose–volume parameters of BMact predict ALCnadir% and PLTnadir% more accurately than BMtot. The employed multivariate regression model effectively predicted lymphocyte nadir (expressed as a percentage of the initial value). Baseline lymphocyte ≤1.83 × 10^3^/ml allowed the prediction of grade 3 CTCAE lymphopenia with high specificity and sensitivity.
